# Glucose becomes one of the worst carbon sources for *E.coli* on poor nitrogen sources due to suboptimal levels of cAMP

**DOI:** 10.1038/srep24834

**Published:** 2016-04-25

**Authors:** Anat Bren, Junyoung O. Park, Benjamin D. Towbin, Erez Dekel, Joshua D. Rabinowitz, Uri Alon

**Affiliations:** 1 Dept. of Molecular Cell Biology, Weizmann Institute of Science, Rehovot Israel 76100; 2 Lewis-Sigler Institute for Integrative Genomics, Princeton University, Princeton, NJ 08544, USA; 3 Department of Chemical and Biological Engineering, Princeton University, Princeton, NJ 08544, USA; 4 Department of Chemistry, Princeton University, Princeton, NJ 08544, USA

## Abstract

In most conditions, glucose is the best carbon source for *E. coli:* it provides faster growth than other sugars, and is consumed first in sugar mixtures. Here we identify conditions in which *E. coli* strains grow slower on glucose than on other sugars, namely when a single amino acid (arginine, glutamate, or proline) is the sole nitrogen source. In sugar mixtures with these nitrogen sources, *E. coli* still consumes glucose first, but grows faster rather than slower after exhausting glucose, generating a reversed diauxic shift. We trace this counterintuitive behavior to a metabolic imbalance: levels of TCA-cycle metabolites including α-ketoglutarate are high, and levels of the key regulatory molecule cAMP are low. Growth rates were increased by experimentally increasing cAMP levels, either by adding external cAMP, by genetically perturbing the cAMP circuit or by inhibition of glucose uptake. Thus, the cAMP control circuitry seems to have a ‘bug’ that leads to slow growth under what may be an environmentally rare condition.

*E. coli* can utilize many different carbon and nitrogen sources. This generates a large number of possible combinations of nutrients. It is of interest to understand what guides decision making in this landscape of nutrient combinations, and whether the decisions made are optimal in terms of growth.

The preferred carbon source for *E. coli*, as for many other bacteria, is glucose, supporting faster growth rate compared to other sugars. The best known example of preferential glucose utilization comes from the work of Monod on the glucose-lactose diauxic shift: *E. coli* first grows rapidly on glucose, and when glucose runs out shifts to grow more slowly on lactose or other sugars^1^. Glucose prevents the use of other carbon sources, a phenomena termed carbon catabolic repression (CCR)^2^. CCR is believed to be important in natural environments to allow the bacteria to maximize growth rate on its preferred sugar. Glucose prevents the use of other carbon sources by inducer exclusion, and by inhibiting the synthesis of the signaling molecule cAMP. cAMP activates the global transcription factor CRP, which regulates the transcription of more than 100 operons with diverse functions^3^ including alternative sugar utilization systems^4^,^5^,^6^.

The best nitrogen source for *E. coli* is ammonia^7^,^8^. *E. coli* can also grow, albeit more slowly, on a variety of other nitrogen sources including many amino acids^7^.

Carbon and nitrogen metabolism is linked, requiring shared regulation between these two elements. This shared regulation involves a key branch point between carbon and nitrogen utilization pathways in which α-ketoglutarate (αKG) serves both as a TCA intermediate and as the carbon backbone of glutamate and glutamine, the main nitrogen currencies^9^,^10^,^11^. In addition, αKG serves as a regulatory molecule, and together with glutamine it regulates the nitrogen assimilation system^10^,^12^,^13^,^14^,^15^,^16^. Recently it was shown that αKG has a regulatory role also in carbon assimilation^17^,^18^. Thus, under nitrogen limiting conditions which reduce the flux of αKG out of the TCA cycle into amino acids biosynthesis, αKG levels rise, leading to repression of glucose uptake^17^ and inhibition of cAMP synthesis by adenylate cyclase^18^,^19^, providing circuits that coordinate between carbon and nitrogen assimilation^15^,^16^,^20^.

The extent to which these circuits are able to optimize (or nearly optimize) growth rate for different combinations of carbon and nitrogen sources remains an open question. Here, we tested many different combinations of carbon and nitrogen sources, on a wide variety of laboratory and naturally occurring strains. We found that under some poor nitrogen sources such as certain amino acids, glucose becomes one of the worst carbon sources, supporting lower growth rate than other sugars. This is due to very low cAMP levels that are suboptimal for growth: growth can be improved by experimentally increasing cAMP levels, by either adding external cAMP, genetically manipulating cAMP levels, or inhibiting glucose uptake. Despite the low growth rate on glucose, it is utilized first in a mixture with a secondary sugar, resulting in a ‘reversed’ diauxic shift with a second phase that has higher growth rate than the first phase. Mass spectrometry showed that glucose with a poor nitrogen source (arginine) results in high levels of TCA intermediates including αKG, explaining the disadvantageously low cAMP levels in this condition. Thus, the cAMP circuitry seems to have a ‘bug’ that leads to slow growth under what may be an environmentally rare condition.

## Results

### On some amino acids as nitrogen sources, glucose is a worse carbon source than other sugars

We measured the growth rate of *E. coli* NCM3722 (a fully sequenced K12 strain^21^ which lacks some of the loss-of-function mutations of MG1655^22^), on a defined minimal medium with different carbon and nitrogen sources. We used glucose, lactose, arabinose, maltotriose, glycerol, sorbitol, xylose, rhamnose or mannose as carbon sources. Nitrogen sources included saturating ammonia (the best nitrogen source, 18.7 mM) or saturating levels of arginine, proline, aspargine, glutamine, or glutamate (10 mM). We grew bacteria in 96-well plates and measured the optical density every 8 min, in a robotic system^23^. We calculated the growth rate in each condition by the logarithmic derivative of the OD curve at mid exponential phase^24^,^25^.

**Figure 1:**
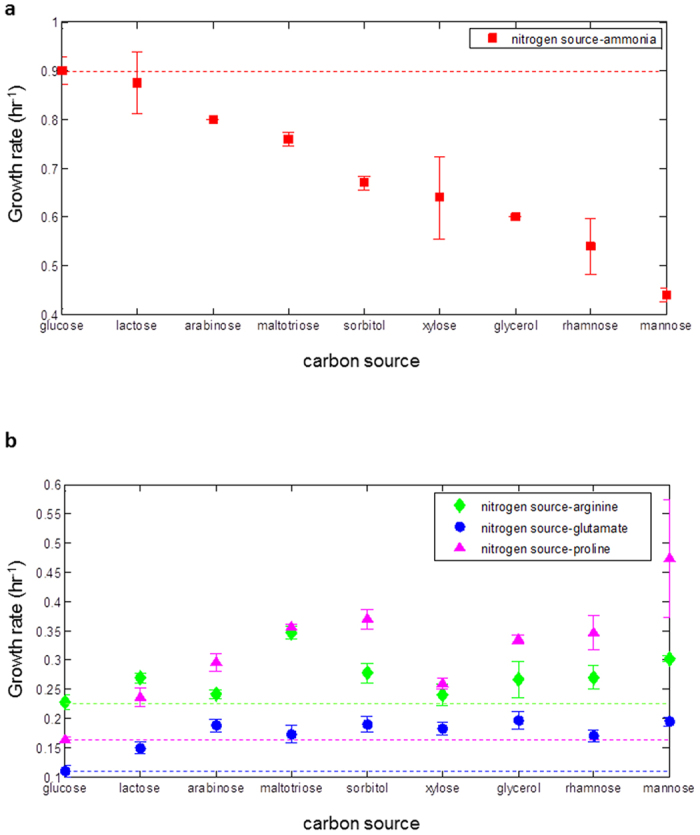
On certain poor nitrogen sources, glucose is no longer the best carbon source. Exponential growth rate of NCM3722 strain on various sugars (0.2%, except maltotriose 0.12%) in (**a**) NH_4_Cl (18.7 mM) as a nitrogen source (**b**) arginine, glutamate, or proline as the nitrogen source (10 mM). Dashed lines represent the growth rate on glucose for each of the nitrogen sources. Growth rate in each condition is the average of 2–4 independent experiments on different days with 6 experimental replicates in each experiment. Error bars are the standard deviation of biological repeats.

When ammonia was used as a nitrogen source, glucose supported the highest growth rate of all sugars, as expected^24^,^26^ (Fig. 1a and SI Table 1), growing 3–105% faster than on other carbon sources. However, with arginine, proline or glutamate as nitrogen sources, glucose resulted in the slowest growth rate (Fig. 1b and SI Table 1), growing 5–36% slower than other carbon sources on arginine, 41–188% slower on proline, and 35–78% slower on glutamate. Not all alternative nitrogen sources showed a growth defect on glucose. Low growth rate on glucose compared to other sugars was not seen for the major amide-donor amino acids (glutamine, asparagine) or for the combination of arginine and glutamate (SI, Table 2). These nitrogen sources supported higher growth rates (at least twice as large) than proline, arginine or glutamate as single nitrogen sources.

To check the strain specificity of this observation, we measured the growth rates of 94 wild *E. coli* strains isolated from different host organisms^27^,^28^ with ammonia, arginine, glutamate or proline as the nitrogen source. With ammonia, glucose supported faster growth than other sugars for all strains. In contrast, we found that for many strains, when the nitrogen source was arginine, glutamate or proline, growth on glucose was slower than on other carbon sources (~20/94 with arginine, ~60/94 with glutamate and ~60/94 with proline, see examples in SI Fig. S1).

### Reversed diauxic-shift in poor nitrogen sources

**Figure 2:**
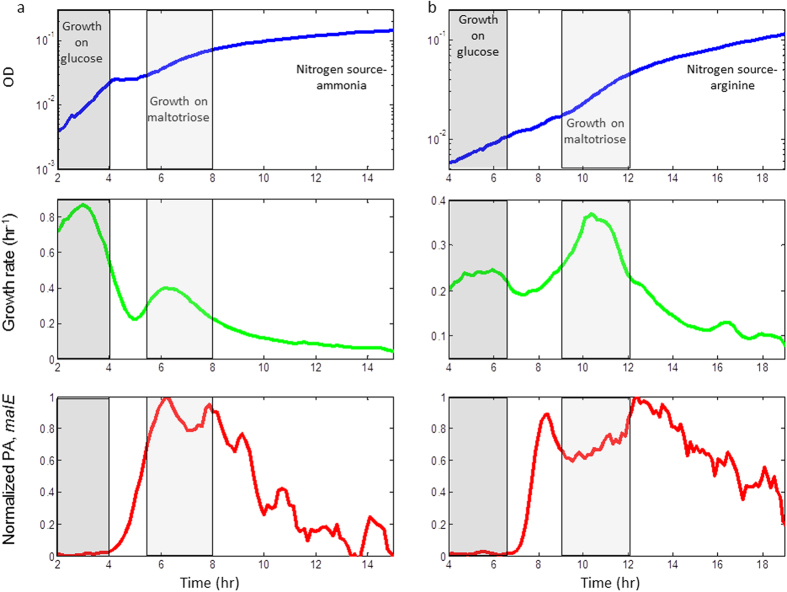
Reversed diauxic shift on a poor nitrogen source. NCM3722 + *malE* reporter was grown in M9 minimal medium containing 0.006% glucose and 0.12% maltotriose with either (**a**) NH_4_Cl (18.7 mM) or (**b**) arginine (10 mM) as a nitrogen source. The upper panels show OD measurements (600 nm) at a temporal resolutions of 8 min, averaged over 48 experimental replicates with standard error of ~2% at each time-point. The middle panels show the instantaneous growth rate (hr^−1^). The lower panels show normalized promoter activity (PA) of the *malE* reporter along the growth curve. Promoter activity was calculated by computing the rate of accumulation of GFP per unit time divided by the OD (dGFP/dt/OD), and was normalized to the maximal value for each curve. Each point in the graph represents the average promoter activity of 48 experimental replicates with standard error of ~3% at each time-point. Growth phases (shaded) were defined by the points at which growth rate goes halfway between the adjacent maximum and minimum levels.

The finding that under certain poor nitrogen sources, secondary carbon sources support higher growth rate than glucose, raises the question whether diauxic shift can still be observed under these conditions. For this purpose we grew bacteria on a mixture of low glucose concentration (0.006%) and a saturating level of a secondary sugar. We chose maltotriose (0.12%) as the second sugar since growth rate on maltotriose with arginine as a nitrogen source was the highest among all sugars tested (Fig. 1b). We measured the growth curve and sugar system promoter activity using a GFP reporter plasmid. With ammonia as the nitrogen source, NCM3722 cells showed the classic diauxic growth curve^1^: they consumed glucose first with a rapid growth rate (0.86 ± 0.02 hr^−1^), and then switched to maltotriose which supported a lower growth rate (0.37 ± 0.005 hr^−1^), accompanied by increased promoter activity of the secondary sugar utilization operon (*malEFG*) (Fig. 2a).

With arginine as a nitrogen source, we also observed two growth phases. However, now the phase with slow growth *precedes* the phase with fast growth (0.24 ± 0.01 hr^−1^ versus 0.36 ± 0.01 hr^−1^) leading to a reversal of the slopes of the growth curve during the diauxic shift. In the absence of the secondary sugar, or in a mutated strain (Δ*malT*) that cannot consume the second sugar, only one phase of growth was obtained, identical to the first phase of the diauxic growth (SI Figs S2, S3). As with ammonia, the *malEFG* reporter is only activated in the second growth phase, indicating that the cells first consume glucose and later maltotriose.

The reversed diauxic-shift phenomenon is also seen with other sugars, provided that the difference in growth rate between glucose and the secondary sugar is large enough (SI Fig. S4). It appears that *E. coli* makes a “wrong” decision by first consuming glucose which provides a slower growth rate than the secondary sugar.

### cAMP-CRP levels are suboptimal for growth on glucose with a poor nitrogen source

Based on findings that the signaling molecule cAMP coordinates carbon and nitrogen utilization^18^, we next asked whether CRP-cAMP is involved in the reversal of growth rates in poor nitrogen.

**Figure 3:**
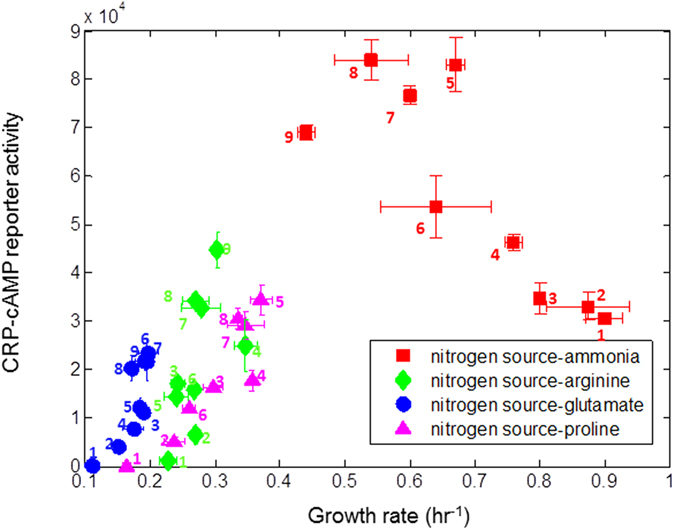
On poor nitrogen sources with glucose, CRP-cAMP activity is very low and the correlation between growth rate and CRP-cAMP activity is positive. Promoter activity of a synthetic reporter responsive to CRP-cAMP in NCM3722 is plotted against the corresponding growth rate in different nitrogen (18.7 mM ammonia or 10 mM of the amino acids) and carbon sources. Carbon sources are labeled by numbers: 1 gluose (0.2%), 2 lactose (0.2%), 3 arabinose (0.2%), 4 maltotriose (0.12%), 5 sorbitiol (0.2%), 6 xylose (0.2%) 7 glycerol (0.2%), 8 rhamnose (0.2%), 9 mannose (0.2%). The numbers are ordered according to the growth rate supported by each carbon source with ammonia as the nitrogen source. Each point is the average promoter activity and growth rate of 2–4 independent experiments on different days with 6 experimental replicates in each experiment. Error bars are the standard deviation of biological repeats.

We measured the promoter activity of a synthetic CRP-cAMP reporter (see methods) in glucose with different nitrogen sources (18.7 mM ammonia, 10 mM proline, 10 mM arginine, 10 mM glutamate). We found that CRP-cAMP reporter activity is unusually low on glucose with poor nitrogen sources. On arginine it is about 30-fold lower than on ammonia, and on glutamate or proline CRP-cAMP promoter activity was not detectable above background in our assay (Fig. 3).

We next measured the relationship between CRP-cAMP activity and growth rate in different carbon and nitrogen sources. With ammonia as a nitrogen source, CRP-cAMP activity is inversely related to growth rate on different carbon sources, in accordance with previous studies^18^,^24^,^26^ (Fig. 3). However, on poor nitrogen sources (arginine, glutamate or proline), we found that the relation is the opposite: for most of the studied sugars the higher the growth rate, the higher the CRP-cAMP reporter activity (Fig. 3, SI fig. S5). This correlation is only approximate, and other factors must contribute to growth rate because in several cases (*e.g.* glucose, lactose) similar CRP-cAMP activity corresponded to different growth rates in different nitrogen sources. In general, we conclude that on the poor nitrogen sources considered here, secondary sugars that produce higher cAMP levels support a higher growth rate compared to “better” sugars that produce lower cAMP levels.

**Figure 4:**
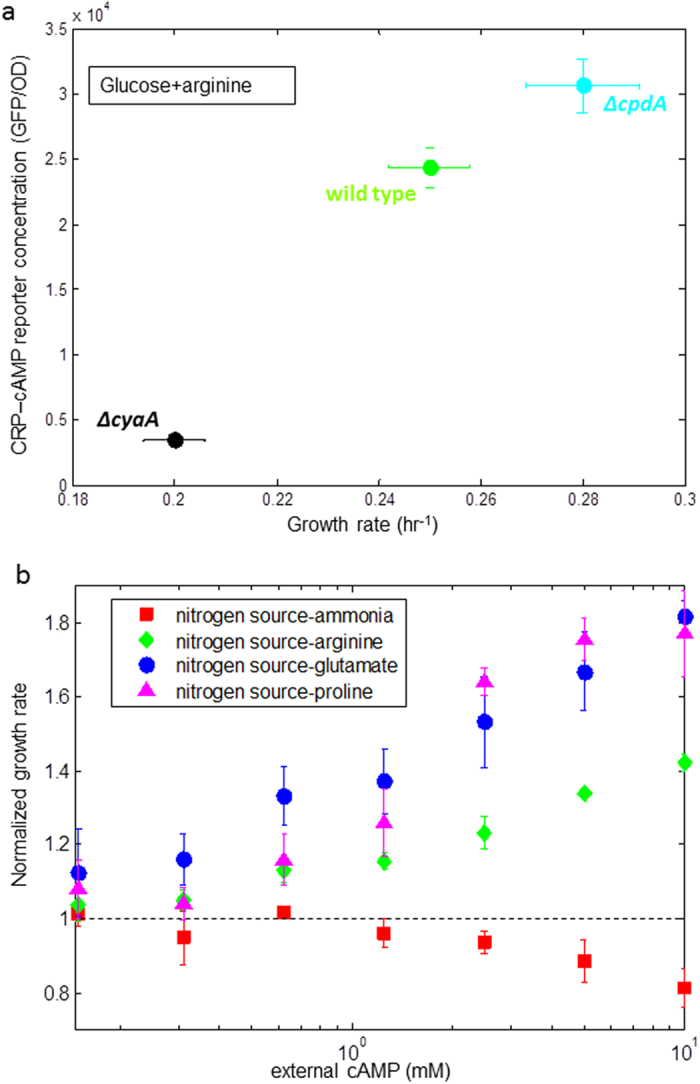
Growth on glucose and a poor nitrogen sources can be improved by increasing cAMP levels by genetic means or external supplement. (**a**) Different mutant strains with glucose as a carbon source (0.2%) and arginine (10 mM) as a nitrogen source. Reporter concentration (GFP/OD) and growth rate (hr^−1^) were determined for the same time window at mid exponential phase. Each point is the average of 3 independent experiments with 6 experimental replicates in each experiment. Due to very low GFP levels for the Δ*cyaA* strain promoter activity was very noisy and we therefore show GFP per OD unit instead of promoter activity (**b**) Exponential growth rate of NCM3722 strain in glucose (0.2%), with different nitrogen sources (18.7 mM ammonia or 10 mM of the amino acids) and externally added cAMP in the indicated levels, normalized to growth rate in the absence of cAMP. Growth rate in each condition is the average of 2–4 independent experiments on different days with 6 experimental replicates in each experiment. Error bars are the standard deviation of biological repeats.

To explore the impact of cAMP on growth rate, we compared the NCM3722 wild-type strain to a mutant strain deleted for the enzyme that synthesizes cAMP (Δ*cyaA*) as well as to a mutant strain deleted for the enzyme that degrades cAMP (Δ*cpdA*). We introduced the CRP reporter plasmid into these strains and measured CRP-cAMP reporter concentration as well as growth rate in glucose+arginine. CRP-cAMP level was indeed reduced in the Δ*cyaA* strain which also showed lower growth rate. The Δ*cpdA* strain had increased levels of CRP-cAMP, accompanied by increased growth rate (Fig. 4a).

To further investigate the role of CRP-cAMP on growth rate, we supplied the cells with external cAMP. We found that growth rate on glucose with poor nitrogen sources was significantly improved with external cAMP (Fig. 4b). Cells on glucose+arginine grew more than 40% faster with 10 mM external cAMP, and on glucose+glutamate or glucose+proline, growth was improved by more than 80% compared to growth rate without external cAMP (Fig. 4b). We excluded the possibility that growth improvement was due to usage of cAMP as an alternative nitrogen source, since external cAMP did not support growth in the absence of an additional nitrogen source. In contrast, on rich nitrogen (ammonia), external cAMP reduced growth rate (Fig. 4b), presumably due to the burden of unnecessary proteins^29^,^30^,^31^ or other toxicity effects. We concluded that on glucose and poor nitrogen, the endogenous level of cAMP is suboptimal for growth.

We next asked whether the low growth rate on glucose+proline/arginine/glutamate might be advantageous in other respects, e.g. by increasing stress resistance or enhancing final yield^32^,^33^. The rationale for this question is that low growth often correlates with high stress tolerance in other contexts^33^. We studied the resistance of *E. coli* NCM3722 to a challenge of H_2_O_2_ (30 mM, see methods). We found that addition of cAMP to cells growing on poor nitrogen (arginine) and glucose, improved not only growth but also survival to the peroxide treatment (SI Fig. S6). This may be due to cAMP up-regulation of survival genes^34^. Moreover, addition of cAMP did not affect final yield (SI Fig. S7)

### Metabolite profiling reveals accumulation of TCA intermediates in glucose and poor nitrogen

**Figure 5:**
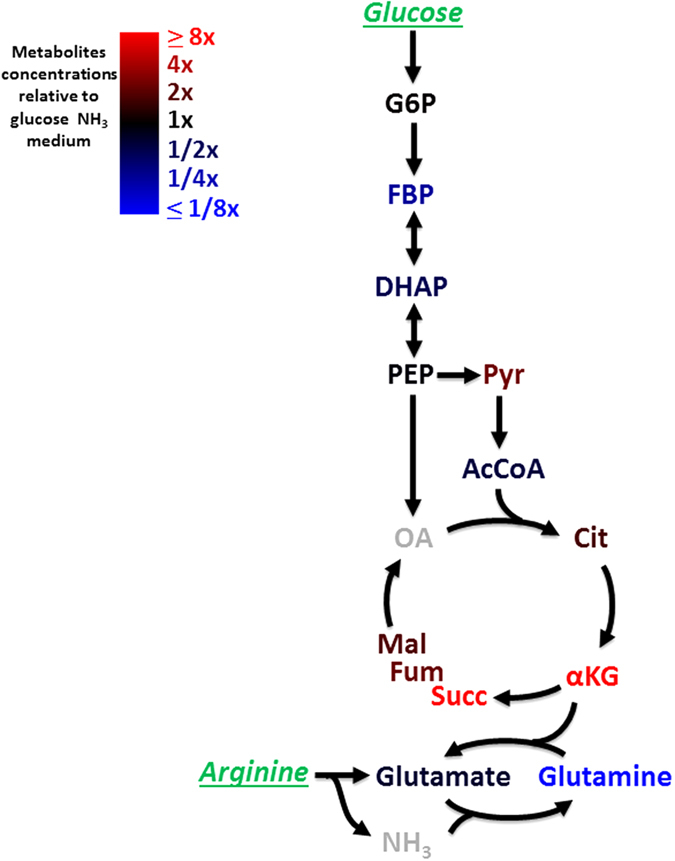
TCA intermediates accumulate on glucose and a poor nitrogen source. Cellular metabolite abundances in glucose (0.4%) + arginine (4.6 mM) minimal medium relative to glucose (0.4%) + ammonia (10 mM) minimal medium. Red indicates metabolite accumulation in arginine culture and blue indicates that metabolite level is lower in arginine culture. Glucose and arginine, which were introduced in the medium, are in green.

In order to trace the metabolic changes which may lead to the non-optimal growth on glucose and the studied poor nitrogen sources, we used mass spectrometry to measure metabolite concentrations on glucose with either ammonia or arginine as nitrogen sources. Exponentially growing cells were quickly vacuum-filtered and brought into extraction solvent for LC-MS metabolomics analysis (see methods). We found that arginine led to an increase in all TCA intermediates compared to ammonia (Fig. 5). Most prominently, αKG levels were more than 10 fold higher, whereas glutamine levels were almost 20 fold lower than on ammonia (Fig. 5, and SI Table S3). This is in line with the reduced activity of the pathways from αKG to glutamine which require free nitrogen which is limiting under the studied conditions.

Nitrogen-source-dependent alteration in TCA intermediates is much milder on glycerol as a carbon source (SI Fig. S8 Table S3). This supports the possibility that inhibition of cAMP synthesis by TCA intermediates such as αKG^18^ underlies the low cAMP level which leads to slow growth under the studied conditions.

It should be noted that other metabolites such as fructose bi-phosphate (FBP) and PEP to pyruvate ratio showed altered levels albeit much less prominent than TCA intermediates (Fig. 5), and may contribute to low cAMP levels^35^ and reduced growth rate under the studied conditions.

### Growth on glucose and poor nitrogen source is improved by partial glucose uptake inhibition, accompanied by increased CRP-cAMP activity and reduced TCA intermediates levels

**Figure 6:**
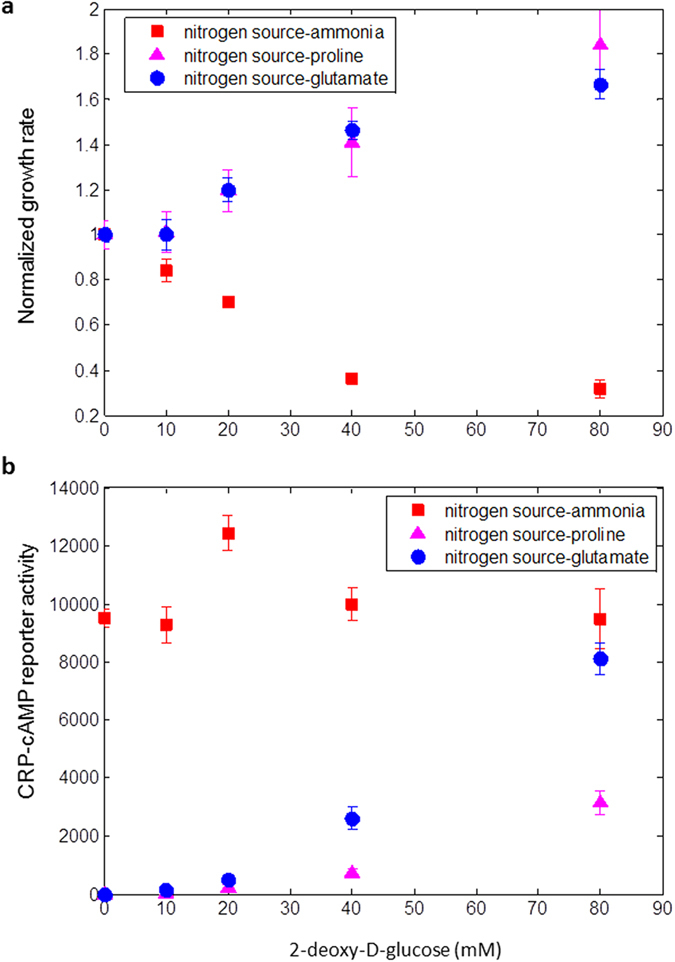
Growth on glucose and a poor nitrogen sources can be improved by glucose uptake inhibition. (**a**) Exponential growth rate of NCM3722 strain in glucose (0.05%), with different nitrogen sources (18.7 mM ammonia or 10 mM of the indicated amino acid) and externally added 2-deoxy-D-glucose. Growth rate in each condition is normalized to growth rate in the absence of the inhibitor. (**b**) Promoter activity of a synthetic GFP reporter responsive to CRP-cAMP with different nitrogen sources and externally added 2-deoxy-D-glucose in the indicated levels. Each point is the average promoter activity and growth rate of 3 independent experiments with 6 experimental replicates in each experiment. Error bars are the standard deviation of biological repeats.

We hypothesized that alleviating carbon-nitrogen imbalance would result in reduced levels of TCA intermediates and hence to increased cAMP levels and improved growth rate. To test this hypothesis, we used two glucose uptake inhibitors, 2-deoxy-D-glucose and methyl α-D-glucopyranoside to reduce glucose influx^36^. With ammonia as a nitrogen source and glucose as a carbon source, growth rate is significantly reduced with increasing inhibitor levels (Fig. 6a, S9). In contrast, with proline or glutamate as nitrogen sources growth rate was improved in the presence of the inhibitors (81% and 66% improvement respectively for 2-deoxy-D-glucose, and 60% and 40% improvement respectively for methyl α-D-glucopyranoside, Fig. 6a and S9). The growth rate increase caused by the inhibitors was accompanied by an increase in CRP-cAMP reporter activity (Fig. 6b, S10) and a decrease in αKG levels measured by LC-MS (SI Fig. S11).

We also tested the effect of deleting *ptsG*, the transmembrane domain of the glucose PTS permease^6^. We found that on arginine, proline or glutamate as sole nitrogen sources and glucose as a carbon source growth was 1.5-3 fold higher in the Δ*ptsG* strain than in the wild-type strain (SI Fig. S12).

These findings support the notion that reduced glucose uptake increases growth with the present poor nitrogen sources.

## Discussion

**Figure 7:**
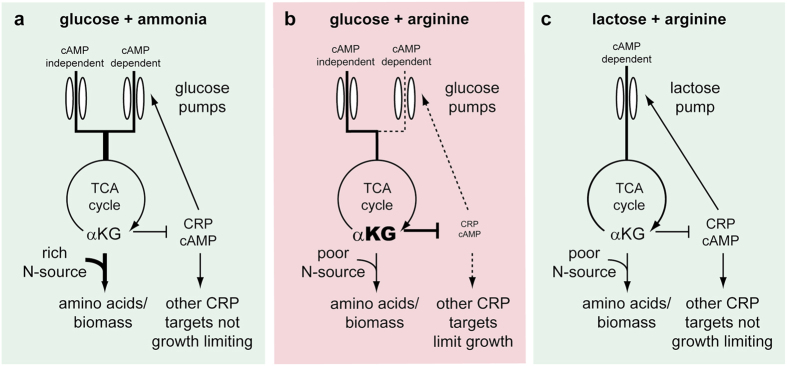
A possible mechanism for slow growth on glucose with poor nitrogen source such as proline, glutamate or arginine. (**a**) On ammonia, TCA cycle intermediates, symbolized here by αKG, are consumed rapidly for amino acid biosynthesis. (**b**) On proline, glutamate or arginine, TCA cycle intermediates are consumed more slowly because of nitrogen shortage. With glucose as carbon source TCA intermediates, symbolized here by αKG build up to high levels which inhibit cAMP levels. cAMP levels fall so low that important genes under control of cAMP-CRP are expressed in inappropriate levels leading to growth limitation. Importantly, uptake of glucose continues even under very low cAMP levels. (**c**) For other carbon sources, such as lactose, cAMP-independent uptake does not take place and their uptake is reduced severely when cAMP levels drop: this creates a feedback control system that prevents cAMP levels from going too low.

We find that glucose, usually one of the best carbon sources for *E. coli*, becomes one of the worst on poor nitrogen sources such as the amino acids arginine, proline or glutamate, supporting lower growth rates than secondary sugars. In diauxic shift experiments on these poor nitrogen sources, glucose is still consumed first but supports a lower growth rate than secondary sugars, resulting in a reversed diauxic shift. The poor growth on glucose appears to arise from carbon/nitrogen imbalance due to excessive levels of αKG which result in insufficient cAMP (Fig. 7). Experimental increase of cAMP by external supply, genetic means or inhibition of glucose influx, leads to faster growth without noticeable negative effects on stress survival or final yield.

This study relates to optimality, a useful perspective for understanding biological regulation. Optimality considerations have led to insights into many aspects of biology^37^ including metabolism^8^,^38^,^39^,^40^ and protein expression^30^,^41^,^42^,^43^. Near-optimal regulation is to be expected for important selectable outputs (e.g., growth, survival) in natural environmental contexts. As highlighted by the present study, however, regulation that may be beneficial in most natural conditions can backfire in other conditions. Here, cells apparently lack the ability to properly stop consuming glucose even when their TCA intermediates are very high, unlike other sugars in which fail-safe mechanisms are in place. Specifically, cells may be unprepared for exponential growth in glucose plus a single amino acid as nitrogen source. Such conditions may be rarely encountered in natural environments^44^,^45^, either because exposure to glucose normally occurs in the presence of ample nitrogen sources (e.g. in the upper intestinal tract) or because a single poor nitrogen source is rarely available, and instead a broader mix of amino acids is typically encountered^46^. These types of ‘bugs’ in the regulation of cells might be expected, considering that the number of regulators and regulatory circuits is much smaller than the number of possible combinations of inputs to these circuits^47^,^48^,^49^,^50^. Thus, it may be unfeasible for the cell to compute the optimal response to all possible combinations of conditions.

A pervious example of suboptimal behavior was observed with cells treated with DNA synthesis inhibitors, in which *E. coli* computes a suboptimal number of ribosomes^51^. This miscalculation is due to a ‘look-up table’ regulation, in which ribosome production is determined by sensing the medium conditions rather than the cells actual growth rate. A further example occurs in yeast mutants that compute ribosomal gene expression according to external signals and not according their actual growth rate^52^,^53^,^54^.

These results may be relevant in biotechnological contexts in which bacteria are grown in nitrogen limitation, for example to produce organic solvents^55^, or hydrogen gas^56^,^57^. It would be interesting to test whether growth under these conditions is sub-optimal and can be improved by changing the carbon source or by genetic manipulation. More generally, it is of interest to explore further combinations of conditions in which cells show suboptimal behavior. Such conditions might open new ways to understand the limitations of regulatory circuits.

## Materials and Methods

### Strains and plasmids

Most experiments in this study were carried out using NCM3722 (and its derivatives). Other experiments were carried out with a collection of *E. coli* isolated from different host organisms^27^ (SI section 1). For promoter activity measurements we used reporter plasmids from our comprehensive library of reporter strains^58^. In this library, promoters of interest control a fast folding green fluorescent protein gene optimized for bacteria (GFPmut2) on a low copy plasmid (pSC101 origin). For the current study we transformed selected reporter plasmids to NCM3722. For measuring CRP-cAMP activity we used a synthetic reporter which is based on the *lacZ* promoter with reshuffled LacI binding sites, previously found to be a faithful reporter for CRP-cAMP activity^23^,^24^. In a control experiment, we found that this reporter shows a Michaelis-Menten- like response to external cAMP (SI Fig. S14). We note that a recent study shows that measuring CRP-cAMP activity is correlated with cAMP excretion rate -a proxy for the internal cAMP concentration^18^. Δ*cyaA,* Δ*cpdA,* Δ*malT* Δ*ptsG* strains were constructed by transducing the appropriate deletion into NCM3722 from the Keio knockout collection (derived from the BW25113 strain^59^) by P1 transduction.

### Growth rate and promoter activity measurements

Cells were grown overnight in M9 minimal medium (42 mM Na_2_HPO_4_, 22 mM KH_2_PO_4_, 8.5 mM NaCl, 18.5 mM NH_4_Cl, 2 mM MgSO_4_, 0.1 mM CaCl) containing 11 mM glucose, and 0.05% casamino acids at 37 °C to ensure non limiting conditions for the pre-culture. Using a robotic liquid handler (FreedomEvo, Tecan), 96-well plates were prepared with 150 μl of M9 minimal medium (lacking NH_4_Cl) with the indicated nitrogen and carbon sources. cAMP was included in the medium at the indicated concentrations. The wells were inoculated with bacteria at a 1:500 dilution from the overnight culture. This high dilution minimizes nutrient leftovers from the overnight culture. Wells were covered with 100 μl of mineral oil (Sigma) to prevent evaporation, a step which we previously found not to significantly affect growth^58^, and transferred into an automated incubator. Each experiment included 6 plates, each plate with two different conditions or strains. Experiments on the collection of wild strains were carried out in a different format: each plate contained one medium (specific carbon and nitrogen sources) and 96 different strains (94 from the wild collection, NCM3722 and MG1655). Cells were grown in an automated incubator with shaking (6 hz) at 37 °C for about 20 hours. Every 8 minutes the plate was transferred by a robotic arm into a multi-well fluorimeter (Infinite F200, Tecan) that read optical density (OD, 600 nm) and GFP fluorescence (535 nm).

### Promoter activity and growth rate calculation

Growth rate was calculated as the temporal derivative of the natural logarithm of the OD curves, μ = dln(OD)/dt. Exponential growth rate is the mean over a region of at least 2 generations with a nearly constant growth rate. For promoter activity calculations, background fluorescence was subtracted from GFP measurements using a reporter strain bearing promoterless vector pUA66 as described^58^. Promoter activity was then calculated using the temporal derivative of GFP divided by OD as described^23^.

### Metabolite measurements

Cells were grown in M9 minimal media (without NH_4_Cl) with glucose or glycerol (0.4% w/v) as the carbon source and arginine (4.6 mM) as the nitrogen source. Another culture was grown in labeled Gutnick minimal (0.4% w/v [U-^13^C_6_] glucose and 10 mM NH_4_Cl). Exponentially growing cells were quickly quenched by vacuum filtering the culture on nylon membrane filters and quickly transferred to plates containing pre-cooled (−20 °C) 40:40:20 methanol/acetonitrile/water solvent. OD (600 nm) was measured at the time of quenching. The cells on the quenched filters were extracted for 20 min at −20 °C and thereafter the mixture was centrifuged at 4 °C. The supernatants from one of the unlabeled M9 cultures and the labeled Gutnick culture were mixed and the mixtures were dried under nitrogen gas and reconstituted in HPLC-grade water. These samples were analyzed by reversed-phase ion-pairing chromatography coupled to an Exactive orbitrap mass spectrometer (ThermoFisher) by electrospray ionization in negative ion mode^60^. Metabolites were identified by mass-to-charge ratio and retention time match to authenticated standards. Absolute metabolite concentrations were determined by taking the ratio of unlabeled metabolites coming from M9 cultures to ^13^C-labeled metabolites of known concentrations in Gutnick media cultures^61^ after correcting for the natural abundance of ^13^C, incomplete labeling due to impurities and carbon (CO_2_) fixation, the OD600 of the two cultures, and the supernatant volume ratios.

### Survival assay

Cells were inoculated in the appropriate growth media at high dilution (1:1000–1:5000) and grown at 37 °C to OD~0.1. Cultures were treated with 30 mM H_2_O_2_ for 45 min, serially diluted and plated on LB plates for viable cells counting (colony forming units-CFU). The pre-treated cultures were also plated in order to determine the initial cell number and calculate survival percentage.

## Additional Information

**How to cite this article**: Bren, A. *et al.* Glucose becomes one of the worst carbon sources for *E.coli* on poor nitrogen sources due to suboptimal levels of cAMP. *Sci. Rep.* **6**, 24834; doi: 10.1038/srep24834 (2016).

## Supplementary Material

Supplementary Information

## References

[b1] Monod J. Recherches sur la croissance des cultures bacttriennes., (Hermann and Cie, 1942).

[b2] Magasanik B. Catabolite repression. Cold Spring Harb Symp Quant Biol 26, 249–256 (1961). 14468226 10.1101/sqb.1961.026.01.031

[b3] Keseler I. M. *et al.* EcoCyc: fusing model organism databases with systems biology. Nucleic Acids Res. 41, D605–12 (2013). 23143106 10.1093/nar/gks1027 PMC3531154

[b4] Gorke B. & Stulke J. Carbon catabolite repression in bacteria: many ways to make the most out of nutrients. Nat Rev Microbiol 6, 613–624 (2008). 18628769 10.1038/nrmicro1932

[b5] Kremling A., Geiselmann J., Ropers D. & de Jong H. Understanding carbon catabolite repression in Escherichia coli using quantitative models. Trends Microbiol. 23, 99–109 (2015). 25475882 10.1016/j.tim.2014.11.002

[b6] Deutscher J., Francke C. & Postma P. W. How phosphotransferase system-related protein phosphorylation regulates carbohydrate metabolism in bacteria. Microbiol. Mol. Biol. Rev. MMBR 70, 939–1031 (2006). 17158705 10.1128/MMBR.00024-06 PMC1698508

[b7] Reitzer L. Nitrogen assimilation and global regulation in Escherichia coli. Annu. Rev. Microbiol. 57, 155–176 (2003). 12730324 10.1146/annurev.micro.57.030502.090820

[b8] Chubukov V., Gerosa L., Kochanowski K. & Sauer U. Coordination of microbial metabolism. Nat. Rev. Microbiol. 12, 327–340 (2014). 24658329 10.1038/nrmicro3238

[b9] Yuan J. *et al.* Metabolomics-driven quantitative analysis of ammonia assimilation in E. coli. Mol. Syst. Biol. 5, n/a–n/a (2009). 10.1038/msb.2009.60 PMC2736657 19690571

[b10] Jiang P., Peliska J. A. & Ninfa A. J. The Regulation of Escherichia coli Glutamine Synthetase Revisited: Role of 2-Ketoglutarate in the Regulation of Glutamine Synthetase Adenylylation State. Biochemistry (Mosc.) 37, 12802–12810 (1998). 10.1021/bi980666u 9737857

[b11] Ikeda T. P., Shauger A. E. & Kustu S. Salmonella typhimuriumApparently Perceives External Nitrogen Limitation as Internal Glutamine Limitation. J. Mol. Biol. 259, 589–607 (1996). 8683567 10.1006/jmbi.1996.0342

[b12] Jiang P., Mayo A. E. & Ninfa A. J. Escherichia coli glutamine synthetase adenylyltransferase (ATase, EC 2.7.7.49): kinetic characterization of regulation by PII, PII-UMP, glutamine, and alpha-ketoglutarate. Biochemistry (Mosc.) 46, 4133–4146 (2007). 10.1021/bi0620510 17355125

[b13] Ninfa A. J. & Atkinson M. R. PII signal transduction proteins. Trends Microbiol. 8, 172–179 (2000). 10754576 10.1016/s0966-842x(00)01709-1

[b14] Hart Y. *et al.* Robust control of nitrogen assimilation by a bifunctional enzyme in E. coli. Mol. Cell 41, 117–127 (2011). 21211727 10.1016/j.molcel.2010.12.023

[b15] Maheswaran M. & Forchhammer K. Carbon-source-dependent nitrogen regulation in Escherichia coli is mediated through glutamine-dependent GlnB signalling. Microbiology 149, 2163–2172 (2003). 12904556 10.1099/mic.0.26449-0

[b16] Commichau F. M., Forchhammer K. & Stülke J. Regulatory links between carbon and nitrogen metabolism. Curr. Opin. Microbiol. 9, 167–172 (2006). 16458044 10.1016/j.mib.2006.01.001

[b17] Doucette C. D., Schwab D. J., Wingreen N. S. & Rabinowitz J. D. α-Ketoglutarate coordinates carbon and nitrogen utilization via enzyme I inhibition. Nat. Chem. Biol. 7, 894–901 (2011). 22002719 10.1038/nchembio.685 PMC3218208

[b18] You C. *et al.* Coordination of bacterial proteome with metabolism by cyclic AMP signalling. Nature 500, 301–6 (2013). 23925119 10.1038/nature12446 PMC4038431

[b19] Daniel J. & Danchin A. 2-Ketoglutarate as a possible regulatory metabolite involved in cyclic AMP-dependent catabolite repression in Escherichia coli K12. Biochimie 68, 303–310 (1986). 3015255 10.1016/s0300-9084(86)80027-x

[b20] Mao X.-J., Huo Y.-X., Buck M., Kolb A. & Wang Y.-P. Interplay between CRP-cAMP and PII-Ntr systems forms novel regulatory network between carbon metabolism and nitrogen assimilation in Escherichia coli. Nucleic Acids Res. 35, 1432–1440 (2007). 17284458 10.1093/nar/gkl1142 PMC1865078

[b21] Brown S. D. & Jun S. Complete Genome Sequence of *Escherichia coli* NCM3722. Genome Announc. 3, e00879–15 (2015). 26251500 10.1128/genomeA.00879-15 PMC4541272

[b22] Soupene E. *et al.* Physiological Studies of Escherichia coli Strain MG1655: Growth Defects and Apparent Cross-Regulation of Gene Expression. J. Bacteriol. 185, 5611–5626 (2003). 12949114 10.1128/JB.185.18.5611-5626.2003 PMC193769

[b23] Kaplan S., Bren A., Zaslaver A., Dekel E. & Alon U. Diverse two-dimensional input functions control bacterial sugar genes. Mol. Cell 29, 786–792 (2008). 18374652 10.1016/j.molcel.2008.01.021 PMC2366073

[b24] Aidelberg G. *et al.* Hierarchy of non-glucose sugars in Escherichia coli. BMC Syst. Biol. 8, 1 (2014). 25539838 10.1186/s12918-014-0133-z PMC4304618

[b25] Bren A., Hart Y., Dekel E., Koster D. & Alon U. The last generation of bacterial growth in limiting nutrient. BMC Syst. Biol. 7, 27 (2013). 23531321 10.1186/1752-0509-7-27 PMC3626568

[b26] Bettenbrock K. *et al.* Correlation between growth rates, EIIACrr phosphorylation, and intracellular cyclic AMP levels in Escherichia coli K-12. J. Bacteriol. 189, 6891–900 (2007). 17675376 10.1128/JB.00819-07 PMC2045212

[b27] Souza V., Rocha M., Valera A. & Eguiarte L. E. Genetic Structure of Natural Populations of Escherichia coli in Wild Hosts on Different Continents. Appl. Environ. Microbiol. 65, 3373–3385 (1999). 10427022 10.1128/aem.65.8.3373-3385.1999 PMC91507

[b28] Razo-Mejia M. *et al.* Comparison of the theoretical and real-world evolutionary potential of a genetic circuit. Phys. Biol. 11, 026005 (2014). 24685590 10.1088/1478-3975/11/2/026005 PMC4051709

[b29] Dong H., Nilsson L. & Kurland C. G. Gratuitous overexpression of genes in Escherichia coli leads to growth inhibition and ribosome destruction. J. Bacteriol. 177, 1497–1504 (1995). 7883706 10.1128/jb.177.6.1497-1504.1995 PMC176765

[b30] Dekel E. & Alon U. Optimality and evolutionary tuning of the expression level of a protein. Nature 436, 588–592 (2005). 16049495 10.1038/nature03842

[b31] Koch A. L. Why can’t a cell grow infinitely fast? Can J Microbiol 34, 421–426 (1988). 2460206 10.1139/m88-074

[b32] Schuetz R., Zamboni N., Zampieri M., Heinemann M. & Sauer U. Multidimensional Optimality of Microbial Metabolism. Science 336, 601–604 (2012). 22556256 10.1126/science.1216882

[b33] Shoval O. *et al.* Evolutionary trade-offs, Pareto optimality, and the geometry of phenotype space. Science 336, 1157–1160 (2012). 22539553 10.1126/science.1217405

[b34] Schultz J. E., Latter G. I. & Matin A. Differential regulation by cyclic AMP of starvation protein synthesis in Escherichia coli. J. Bacteriol. 170, 3903–3909 (1988). 2842291 10.1128/jb.170.9.3903-3909.1988 PMC211388

[b35] Hogema B. M. *et al.* Inducer exclusion in Escherichia coli by non-PTS substrates: the role of the PEP to pyruvate ratio in determining the phosphorylation state of enzyme IIAGlc. Mol. Microbiol. 30, 487–498 (1998). 9822815 10.1046/j.1365-2958.1998.01053.x

[b36] Wick A. N., Drury D. R., Nakada H. I. & Wolfe J. B. Localization of the primary metabolic block produced by 2-deoxyglucose. J. Biol. Chem. 224, 963–969 (1957). 13405925

[b37] Alexander R. M. Optima for Animals. (Princeton University Press, 1996).

[b38] Ibarra R. U., Edwards J. S. & Palsson B. O. Escherichia coli K-12 undergoes adaptive evolution to achieve *in silico* predicted optimal growth. Nature 420, 186–189 (2002). 12432395 10.1038/nature01149

[b39] Lewis N. E. *et al.* Omic data from evolved E. coli are consistent with computed optimal growth from genome-scale models. Mol. Syst. Biol. 6, 390 (2010). 20664636 10.1038/msb.2010.47 PMC2925526

[b40] Goyal S., Yuan J., Chen T., Rabinowitz J. D. & Wingreen N. S. Achieving optimal growth through product feedback inhibition in metabolism. Plos Comput. Biol. 6, e1000802 (2010). 20532205 10.1371/journal.pcbi.1000802 PMC2880561

[b41] Lang G. I., Murray A. W. & Botstein D. The cost of gene expression underlies a fitness trade-off in yeast. Proc. Natl. Acad. Sci. 106, 5755–5760 (2009). 19299502 10.1073/pnas.0901620106 PMC2658138

[b42] Poelwijk F. J., Heyning P. D., de Vos M. G. J., Kiviet D. J. & Tans S. J. Optimality and evolution of transcriptionally regulated gene expression. BMC Syst. Biol. 5, 128 (2011). 21846366 10.1186/1752-0509-5-128 PMC3182923

[b43] Chubukov V., Zuleta I. A. & Li H. Regulatory architecture determines optimal regulation of gene expression in metabolic pathways. Proc. Natl. Acad. Sci. 109, 5127–5132 (2012). 22416120 10.1073/pnas.1114235109 PMC3324031

[b44] Savageau M. A. Escherichia coli Habitats, Cell Types, and Molecular Mechanisms of Gene Control. Am. Nat. 122, 732–744 (1983).

[b45] Blount Z. D. The natural history of model organisms: The unexhausted potential of E. coli. eLife 4, e05826 (2015). 10.7554/eLife.05826 PMC4373459 25807083

[b46] Chang D.-E. *et al.* Carbon nutrition of Escherichia coli in the mouse intestine. Proc. Natl. Acad. Sci. USA 101, 7427–7432 (2004). 15123798 10.1073/pnas.0307888101 PMC409935

[b47] Price M. N. *et al.* Indirect and suboptimal control of gene expression is widespread in bacteria. Mol. Syst. Biol. 9, 660 (2013). 23591776 10.1038/msb.2013.16 PMC3658271

[b48] Charoensawan V., Wilson D. & Teichmann S. A. Genomic repertoires of DNA-binding transcription factors across the tree of life. Nucleic Acids Res. 38, 7364–7377 (2010). 20675356 10.1093/nar/gkq617 PMC2995046

[b49] Martínez-Antonio A. & Collado-Vides J. Identifying global regulators in transcriptional regulatory networks in bacteria. Curr. Opin. Microbiol. 6, 482–489 (2003). 14572541 10.1016/j.mib.2003.09.002

[b50] Maslov S. & Sneppen K. Specificity and Stability in Topology of Protein Networks. Science 296, 910–913 (2002). 11988575 10.1126/science.1065103

[b51] Bollenbach T., Quan S., Chait R. & Kishony R. Nonoptimal microbial response to antibiotics underlies suppressive drug interactions. Cell 139, 707–18 (2009). 19914165 10.1016/j.cell.2009.10.025 PMC2838386

[b52] Zaman S., Lippman S. I., Schneper L., Slonim N. & Broach J. R. Glucose regulates transcription in yeast through a network of signaling pathways. Mol. Syst. Biol. 5, n/a–n/a (2009). 10.1038/msb.2009.2 PMC2657534 19225458

[b53] Levy S. & Barkai N. Coordination of gene expression with growth rate: A feedback or a feed-forward strategy? FEBS Lett. 583, 3974–3978 (2009). 19878679 10.1016/j.febslet.2009.10.071

[b54] Levy S. *et al.* Strategy of Transcription Regulation in the Budding Yeast. Plos ONE 2, e250 (2007). 17327914 10.1371/journal.pone.0000250 PMC1803021

[b55] Monot F. & Engasser J. M. Production of acetone and butanol by batch and continuous culture of Clostridium acetobutylicum under nitrogen limitation. Biotechnol. Lett. 5, 213–218 (1983).

[b56] Aoyama K., Uemura I., Miyake J. & Asada Y. Fermentative metabolism to produce hydrogen gas and organic compounds in a cyanobacterium, Spirulina platensis. J. Ferment. Bioeng. 83, 17–20 (1997).

[b57] Schütz K. *et al.* Cyanobacterial H2 production — a comparative analysis. Planta 218, 350–359 (2003). 14564521 10.1007/s00425-003-1113-5

[b58] Zaslaver A. *et al.* A comprehensive library of fluorescent transcriptional reporters for Escherichia coli. Nat. Methods 3, 623–628 (2006). 16862137 10.1038/nmeth895

[b59] Baba T. *et al.* Construction of Escherichia coli K-12 in-frame, single-gene knockout mutants: the Keio collection. Mol. Syst. Biol. 2, 2006.0008 (2006). 10.1038/msb4100050 PMC1681482 16738554

[b60] Lu W. *et al.* Metabolomic Analysis via Reversed-Phase Ion-Pairing Liquid Chromatography Coupled to a Stand Alone Orbitrap Mass Spectrometer. Anal. Chem. 82, 3212–3221 (2010). 20349993 10.1021/ac902837x PMC2863137

[b61] Bennett B. D. *et al.* Absolute metabolite concentrations and implied enzyme active site occupancy in Escherichia coli. Nat. Chem. Biol. 5, 593–599 (2009). 19561621 10.1038/nchembio.186 PMC2754216

