# Supplementary material for: Glucose becomes one of the worst carbon sources for E.coli on poor nitrogen sources due to suboptimal levels of cAMP
Source: Sci Rep. 2016 Apr 25;6:24834. doi: 10.1038/srep24834 (PMC4843011; doi:10.1038/srep24834)
Supplement: Supplementary Information [file srep24834-s1.docx]

**Supplementary information**

**Glucose becomes one of the worst carbon sources for *E.coli* on poor nitrogen sources due to suboptimal levels of cAMP**

Anat Bren, Junyoung O. Park, Benjamin D. Towbin, Erez Dekel, Joshua D. Rabinowitz and Uri Alon

**Content**

1. On some poor nitrogen sources glucose becomes the worst carbon source………2
2. Not on all nitrogen sources glucose is the worst carbon source………………………...2
3. On proline as the nitrogen source, glucose is the worst carbon source for many *E. coli* strains…………………………………………………………………………………………….3
4. In low glucose levels (with no secondary sugar), the growth curve obtained is identical to the first phase of the diauxic growth…………………………………………….4
5. In Δ*malT* strain, the growth curve obtained is identical to the first phase of the diauxic growth………………………………………………………………………………………….5
6. Reversed diauxic shift with arabinose and rhamnose as the secondary carbon source…6
7. Correlation between CRP-cAMP activity and growth rate shown for each nitrogen source separately…………………………………………………………………………………7
8. Low cAMP levels were found to deleterious or neutral in two other tasks………..8
9. Accumulation of TCA intermediates in glycerol is less pronounced compared to glucose………………………………………………………………………………………………………….10
10. Absolute concentrations of central metabolites in glucose and glycerol with arginine as a nitrogen source…………………………………………………………………….11
11. Growth on glucose and poor nitrogen source is improved by partial glucose uptake inhibition using methyl α-D-glucopyranoside……………………………12
12. CRP-cAMP reporter activity is increased and α-ketoglutarate levels are reduced in the presence of the glucose uptake inhibitor 2-deoxy-D-glucose……13
13. Growth on glucose and poor nitrogen source is significantly improved in a Δ*ptsG* strain…………………………………………………………………………………………………..14
14. CRP-cAMP reporter shows a Michaelis-Menten- like response to cAMP in a ΔcyaA strain…………………………………………………………………………………………………..14
15. **On some poor nitrogen sources glucose becomes the worst carbon source.**

| Nitrogen source/ Carbon source | Ammonia  (hr^-1^) | Arginine  (hr^-1^) | Glutamate  (hr^-1^) | Proline  (hr^-1^) |
| --- | --- | --- | --- | --- |
| Glucose | 0.9±0.028 | 0.23±0.012 | 0.11±0.01 | 0.163±0.006 |
| Lactose | 0.88±0.064 | 0.27±0.008 | 0.15±0.01 | 0.23±0.015 |
| Arabinose | 0.8±0.01 | 0.24±0.007 | 0.19±0.01 | 0.30±.015 |
| Maltotriose | 0.76±0.014 | 0.35±0.011 | 0.17±0.015 | 0.36±0.006 |
| Sorbitol | 0.67±0.014 | 0.29±0.017 | 0.19±0.014 | 0.37±0.017 |
| Xylose | 0.64±0.85 | 0.24±0.018 | 0.18±0.01 | 0.26±0.01 |
| Glycerol | 0.6±0.01?? | 0.25±0.03 | 0.2±0.015 | 0.34±0.007 |
| Rhamnose | 0.54±0.057 | 0.27±0.02 | 0.17±0.01 | 0.35±0.029 |
| Mannose | 0.44±0.014 | 0.3±0.005 | 0.2±0.007 | 0.47±0.1 |

Table S1. Exponential growth rate of NCM3722 strain on various sugars in NH_4_Cl (18.7mM) arginine (10mM) glutamate (10mM) or proline (10mM) as the nitrogen source with different carbon sources (0.2% each, except maltotriose (0.12%)). Growth rate (hr^-1^) in each condition is the average of 2-4 independent experiments on different days with 6 experimental replicas in each experiment.

1. **Not on all nitrogen sources glucose is the worst carbon source**

| Nitrogen source/ Carbon source | Glutamine  (hr^-1^) | Aspargine  (hr^-1^) | Arginine+Glutamate  (hr^-1^) |
| --- | --- | --- | --- |
| Glucose | \| 0.85±0.035 \| \| --- \| | 0.37±0.05 | 0.34±0.014 |
| Lactose | \| 0.86±0.014 \| \| --- \| | 0.36±0.035 | 0.38±0.014 |
| Arabinose | 0.74±0.028 | 0.4±0.07 | 0.34±0.014 |
| Maltotriose | \| 0.745±0.035 \| \| --- \| | 0.38±0 | 0.38±0.035 |
| Glycerol | 0.63±0 | 0.39±0 | 0.3±0.021 |
| Rhamnose | \| 0.635±0.049 \| \| --- \| | 0.38±0.042 | 0.35±0.028 |

Table S2. Exponential growth rate of NCM3722 strain on various sugars in glutamine (10mM) ,asparagine (10mM) and the combination of arginine and glutamate (10mM each) as the nitrogen source with different carbon sources. Growth rate (hr^-1^) in each condition is the average of 2 independent experiments on different days with 6 experimental replicas in each experiment.

1. **On proline as the nitrogen source, glucose is the worst carbon source for many *E. coli* strains isolated from different host organisms.** As a control experiment for host specificity we grew 94 wild *E. coli* strains from different sources^1^ (strain numbers U1001-1094) in different carbon sources and proline as a nitrogen source. We found that for many of the strains in the collection glucose is still the worst carbon source as demonstrated in Fig. S1.


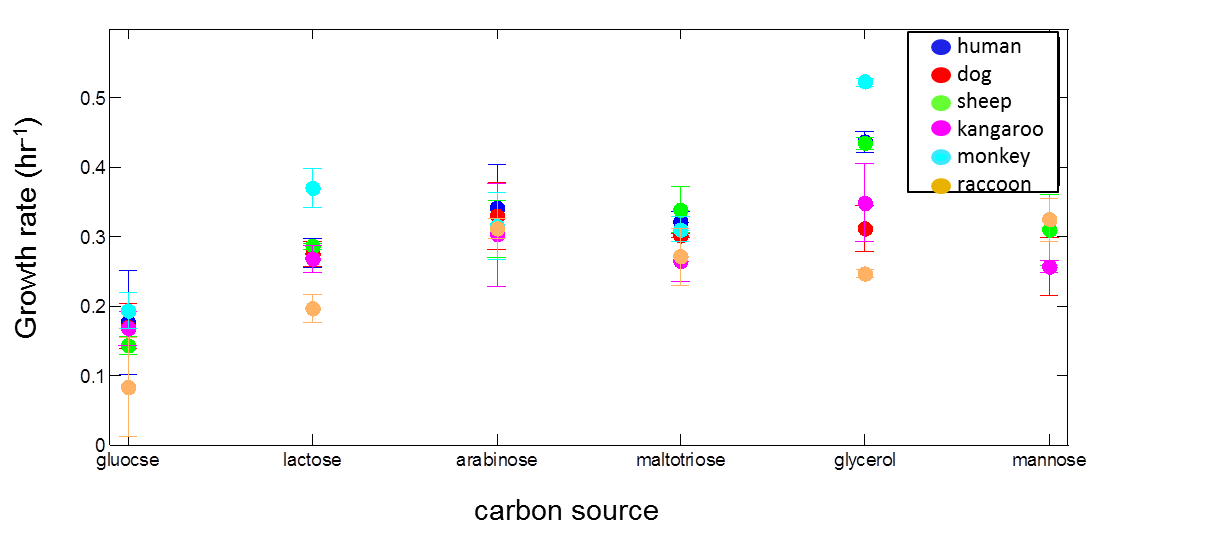


**Fig. S1**. **Exponential growth rate of several *E. coli* strains from different sources with proline (10mM) as the nitrogen source and different carbon source** (0.2% each, except maltotriose (0.12%)). Each point is the average growth rate of 2 independent experiments. Strains designation: human-U1063, dog-U1092, sheep-U1091, kangaroo-U1087, monkey- U1077, raccoon-U1046

1. **In low glucose levels (with no secondary sugar), the growth curve obtained is identical to the first phase of the diauxic growth**. We compared the growth curve on low glucose and saturating maltotriose (Fig. 2b in the main text), to the growth curve on low glucose alone. We found that the growth curve on glucose alone is nearly identical to the first phase of growth on the sugar mixture, supporting the conclusion that glucose is consumed first although it supports lower growth rate.

**Fig. S2. The first phase of growth in diauxic conditions overlaps growth in low glucose alone.** NCM3722+*malE* reporter was grown in M9 minimal medium containing arginine (10mM) as a nitrogen source and 0.006% glucose with or without 0.2 % maltotriose. OD measurements (600 nm) were obtained at a time resolutions of 8 min, averaged over 48 experimental replicas with standard error on the order of ~2% at each time-point.

1. **In Δ*malT* strain, the growth curve obtained is identical to the first phase of the diauxic growth**. We compared the growth curve on low glucose and saturating maltotriose (Fig. 2b in the main text), to the growth curve of a Δ*malT* which cannot grow on maltotriose as a carbon source. We found that the growth curve of the Δ*malT* strain is nearly identical to the first phase of growth of the WT strain.

**
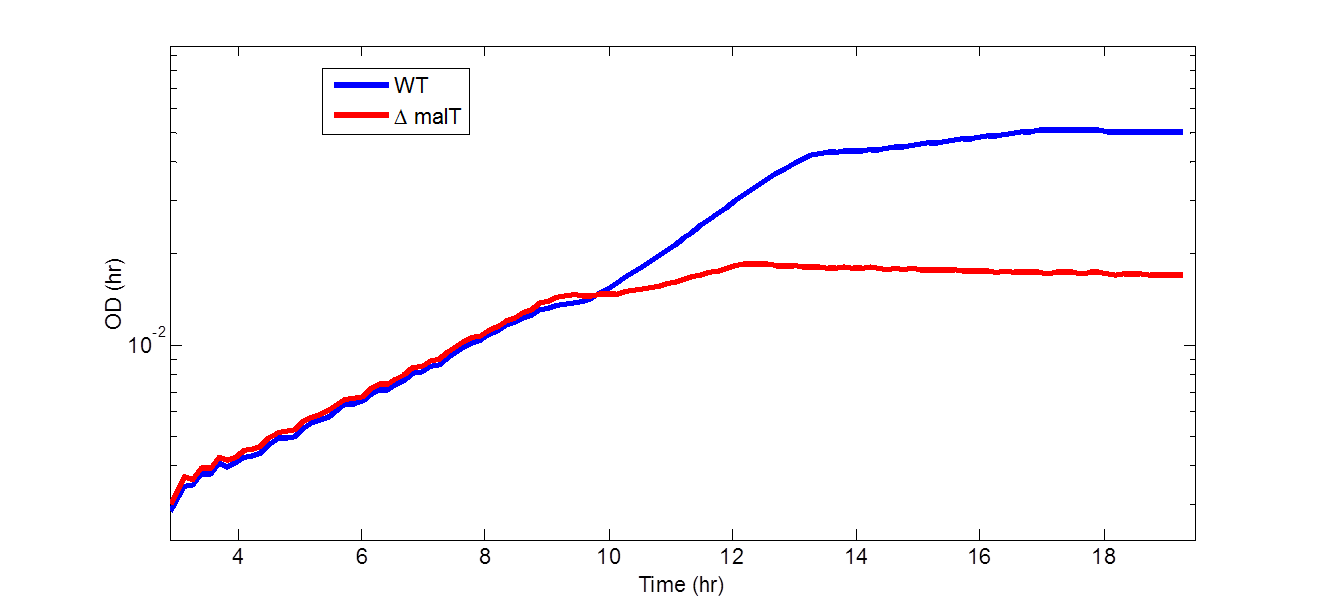
**

Growth rate (hr^-1^)

CRP-cAMP reporter activity

proline

arginine

glutamate

**glucose**

**lactose**

**xylose**

**arabinose**

**sorbitol**

**maltotriose**

**rhamnose**

**glycerol**

**glucose**

**lactose**

**maltotriose**

**glucose**

**lactose**

**xylose**

**arabinose**

**sorbitol**

**maltotriose**

**rhamnose**

**glycerol**

**maltotriose**

**mannose**

**xylose**

**arabinose**

**rhamnose**

**glycerol**

**mannose**

**sorbitol**

**Fig. S3. Growth of a ΔmalT strain under diauxic conditions overlay the first phase of the WT strain.** NCM3722+*malE* reporter was grown in M9 minimal medium containing arginine (10mM) as a nitrogen source and 0.01% glucose +0.2 % maltotriose. OD measurements (600 nm) were obtained at a time resolutions of 8 min, averaged over 48 experimental replicas with standard error on the order of ~2% at each time-point.

1. **Reversed diauxic shift with arabinose and rhamnose as the secondary carbon source.
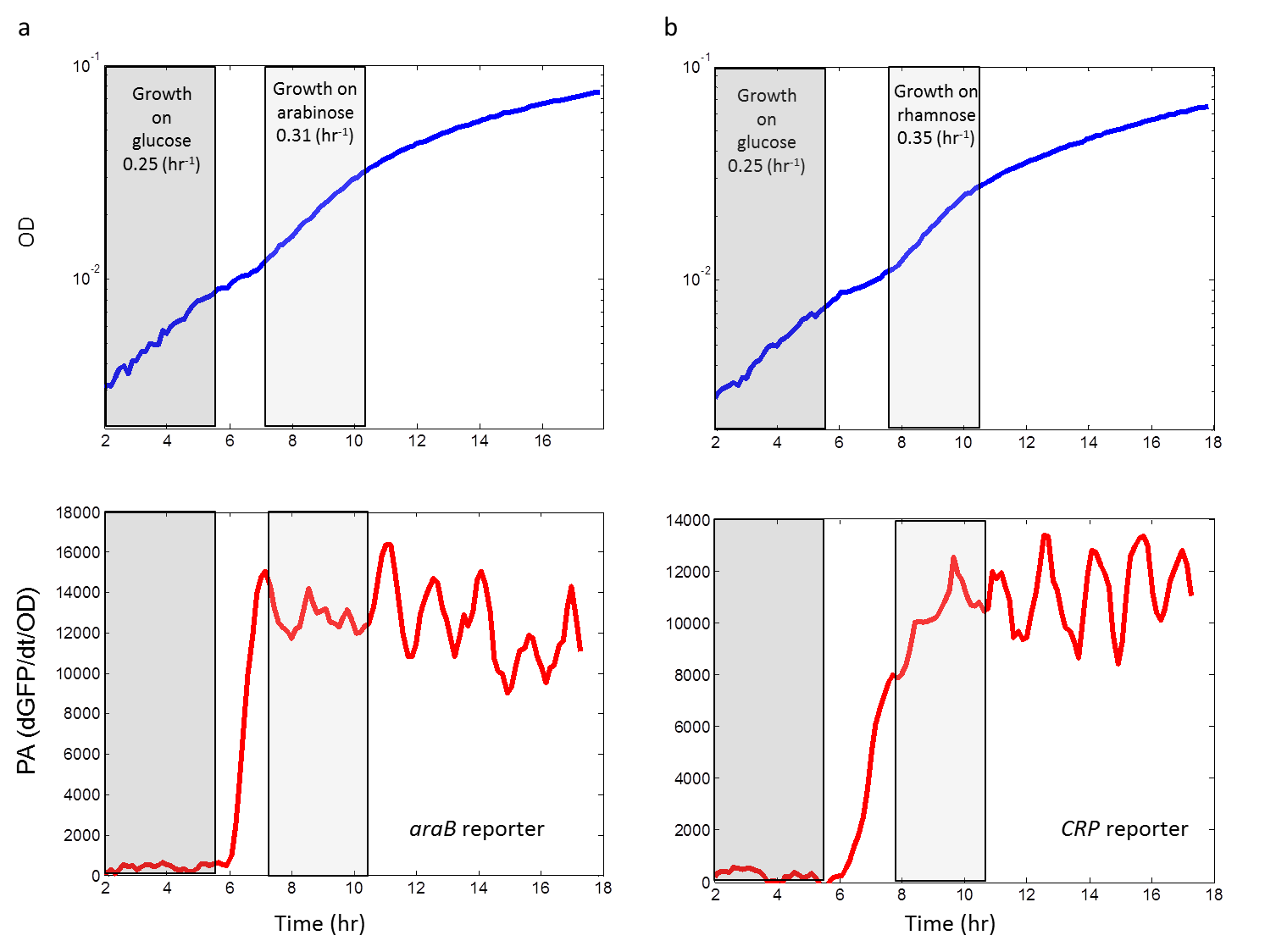
**

**Fig. S4. Reversed diauxic shift on proline as a nitrogen source.** NCM3722+*araB* reporter (a) or CRP-cAMP reporter (b) was grown in M9 minimal medium containing 10mM proline as the nitrogen source with 0.006% glucose and either 0.2% arabinose (a) or 0.2% rhamnose (b). The upper panels show OD measurements (600 nm) at a time resolutions of 8 min, averaged over 48 experimental replicas with standard error on the order of ~2% at each time-point. The lower panels show promoter activity of the indicated reporters along the growth curve. Promoter activity was calculated by computing the rate of accumulation of GFP per unit time divided by OD (dGFP/dt/OD). Each point in the graph represents the average promoter activity of 48 experimental replicas with standard error on the order of ~3% at each time-point.

1. **Correlation between CRP-cAMP activity and growth rate shown for each nitrogen source separately**

**
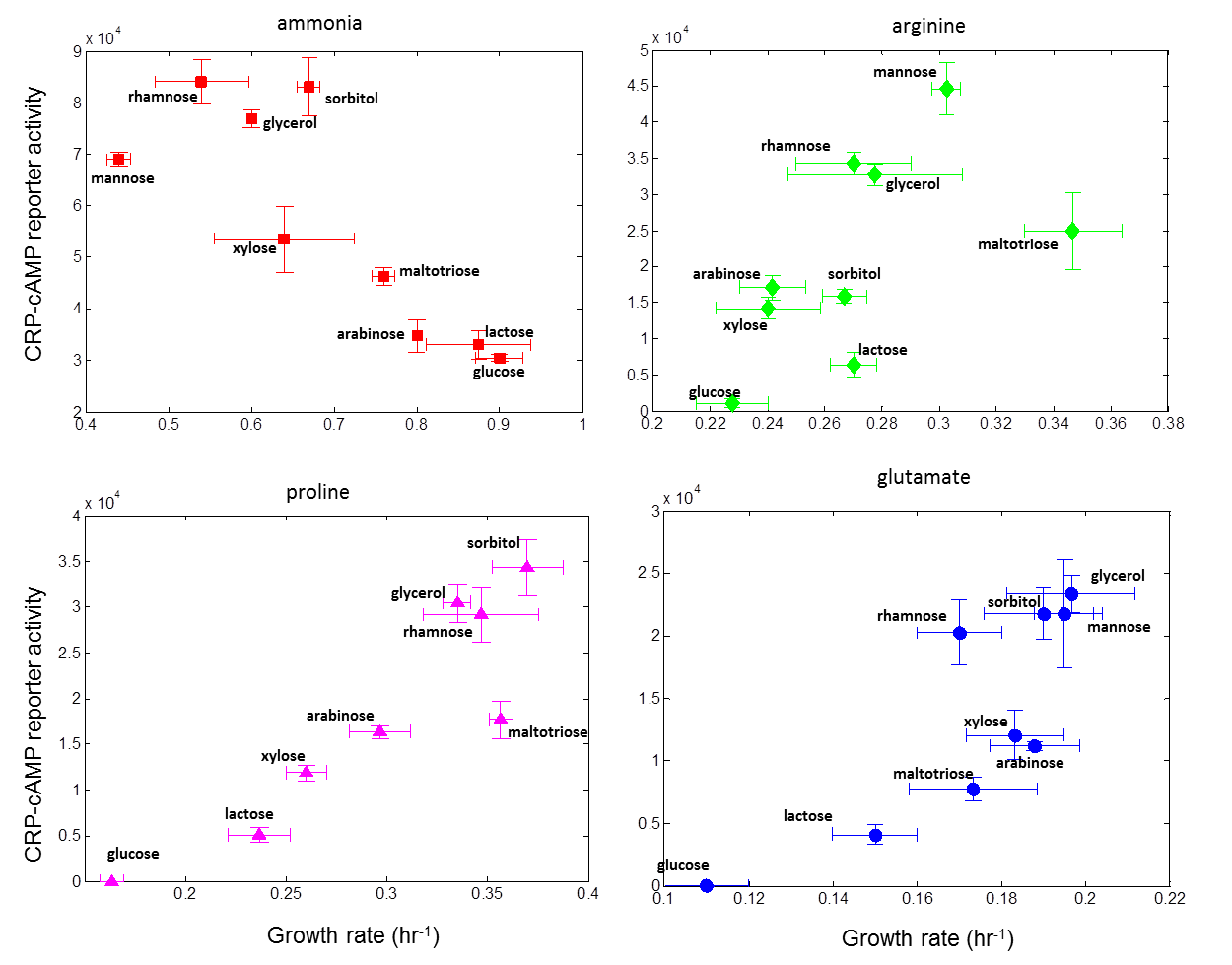
**

**Fig. S5. correlation between CRP-cAMP and growth rate is as expected negative for ammonia but positive for the poor nitrogen sources arginine, proline and glutamate.** Promoter activity of a synthetic CRP-cAMP reporter is plotted against the corresponding growth rate for 9 different carbon sources in different nitrogen sources. Each point is the average promoter activity and growth rate of 2-4 independent experiments on different days with 6 experimental replicates in each experiment. Error bars represents the standard deviation of biological repeats.

1. **Low cAMP levels were found to be deleterious or neutral in two other tasks.** In light of the theory of evolutionary tradeoffs ^2^ we wished to find out whether low cAMP levels which lead to low growth rate are advantageous in other tasks. We tested two tasks: stress resistance and final yield. In both tasks, low cAMP level had no advantage (Figs. S6 and S7 respectively). Moreover stress resistance improved with cAMP, suggesting that ultra-low cAMP levels may be deleterious not only for growth on glucose+arginine, but also to stress survival.


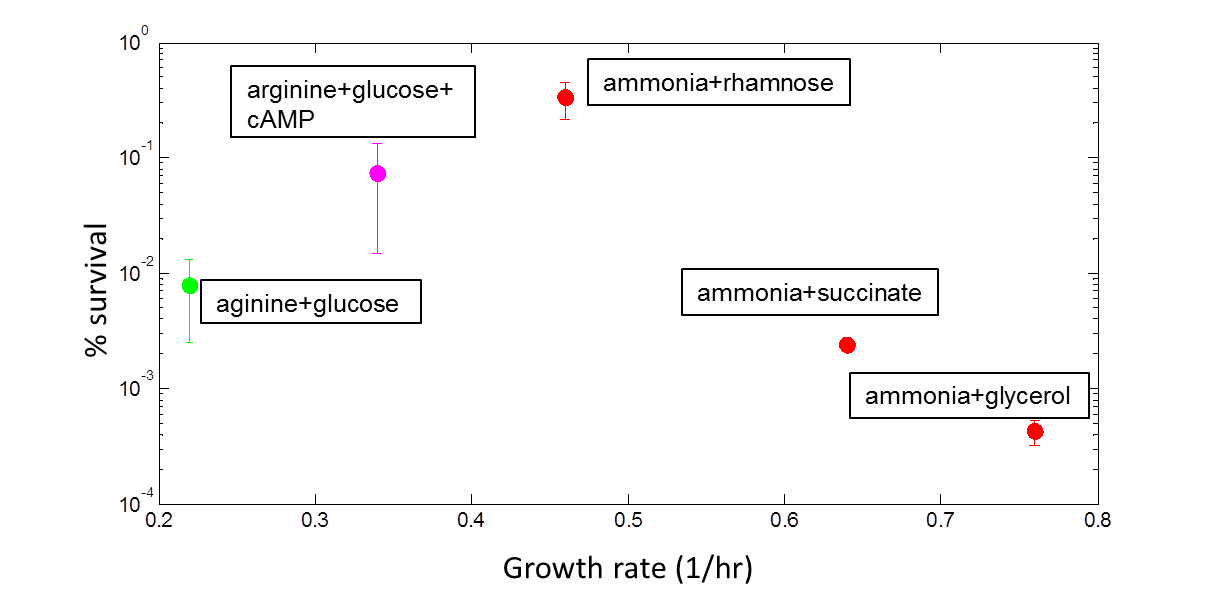


**Fig. S6.** **Low cAMP levels reduce H_2_O_2_ stress survival**. Survivals percentage was determined by colonies count following treatment with 30mM H_2_O_2_ for 45 min. Each point is the average of at least 2 independent experiments. The points for glucose+arginine and glucose+arginine+cAMP are the average of 5 independent experiments.

**Fig.S7.** **Final yield is not strongly dependent on cAMP levels**. NCM3722 was grown in M9 minimal medium containing glucose with arginine (10mM) or glutamate (10mM) as a nitrogen source, in the presence or absence of 10mM cAMP. OD measurements (600 nm) were obtained at a time resolutions of 8 min, averaged over 6 experimental replicas.

1. **Accumulation of TCA intermediates in glycerol is less pronounced compared to glucose**

**≥ 8x**

**4x**

**1x**

**1/2x**

**≤ 1/8x**

**2x**

**1/4x**

**G6P**

**FBP**

**DHAP**

**OA**

**Pyr**

**AcCoA**

**Cit**

**αKG**

**Fum**

**PEP**

**Glutamate**

**Glutamine**

***Arginine***

***Glycerol***

**NH_3_**

**Mal**

**Succ**

Normalized growth rate

methyl α-D-glucopyranoside (mM)

**Fig S8.** **Cellular metabolite abundances in glycerol minimal medium when arginine is the sole nitrogen source.** Metabolite levels are shown relative to those in ammonia and glucose medium. Red indicates metabolite accumulation in glycerol+arginine culture and blue indicates that metabolite level is lower.

1. **Absolute concentrations of central metabolites in glucose and glycerol with arginine as a nitrogen source**

| **metabolite** | **Glucose+Arg (mM)** | **Glycerol+ Arg(mM)** |
| --- | --- | --- |
| hexose-phosphate | 8.7 ± 0.6 | 8.4 ± 0.6 |
| fructose-1,6-bisphosphate | 4.9 ± 0.3 | 3.0 ± 0.3 |
| dihydroxyacetone-phosphate | 1.7 ± 0.1 | 1.4 ± 0.1 |
| 3-phosphoglycerate | 0.9 ± 0.04 | 1.8 ± 0.1 |
| phosphoenolpyruvate | 0.2 ± 0.03 | 0.5 ± 0.06 |
| Pyruvate | 8.9 ± 0.7 | 4.8± 0.5 |
| acetyl-CoA | 0.4 ± 0.07 | 0.3 ± 0.05 |
| Citrate | 3.6 ± 1.1 | 5.0± 1.5 |
| α-ketoglutarate | 13.8 ± 2.8 | 1.2 ± 0.25 |
| succinyl-CoA | 0.14 ± 0.04 | 0.14 ± 0.04 |
| Succinate | 10.2 ± 2.7 | 1.0 ± 0.3 |
| Malate | 3.6 ± 0.1 | 1.2 ± 0.05 |
| Glutamate | 69.4 ± 3.1 | 64.9 ± 2.8 |
| Glutamine | 0.23 ± 0.02 | 0.36 ± 0.03 |
| Aspartate | 1.4 ± 0.22 | 1.5 ± 0.16 |

Table S3. Metabolite level were determined by taking the ratio of each of the metabolite signals in the respective conditions to those in Gutnick glucose NH_3_ minimal media reported by Bennet *et. al*^3^ and Park *et al*. (unpublished results).

1. **Growth on glucose and poor nitrogen source is improved by partial glucose uptake inhibition using methyl α-D-glucopyranoside.**

**
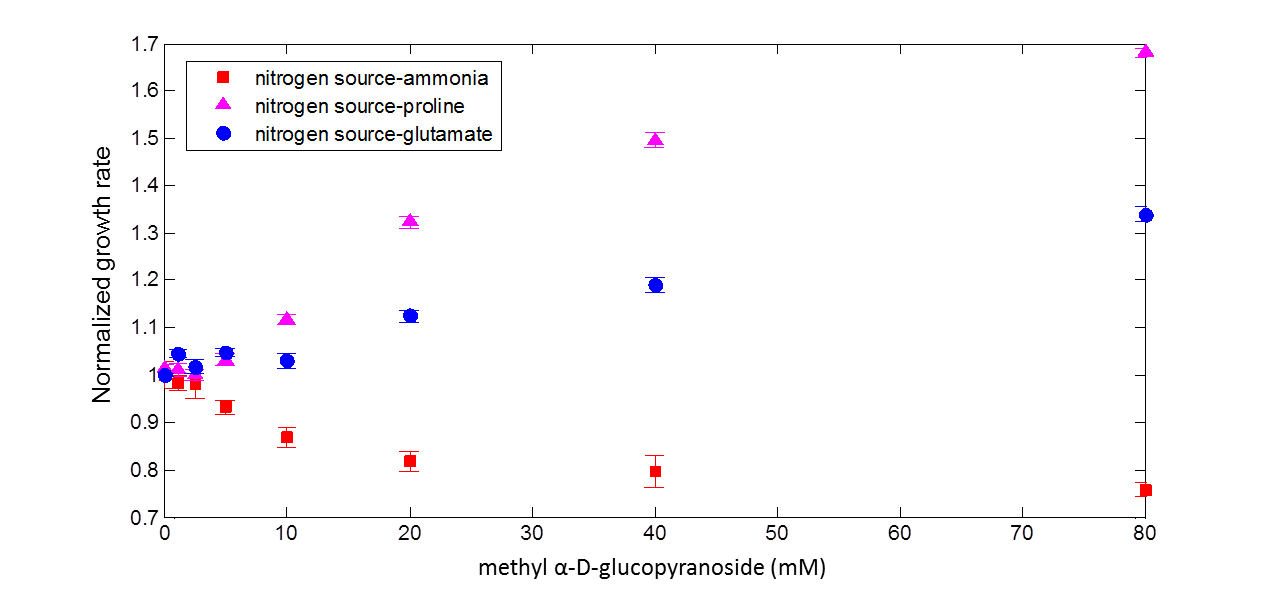
**

**Fig. S9**. **Growth on glucose and a poor nitrogen source is improved by glucose uptake inhibition.** Exponential growth rate of NCM3722 strain in glucose (0.2%), with different nitrogen sources (18.7mM ammonia or 10 mM of the indicated amino acid) and externally supplied methyl α-D-glucopyranoside, normalized to the growth rate in the absence of the inhibitor.

1. **CRP-cAMP reporter activity is increased and α-ketoglutarate levels are reduced in the presence of the glucose uptake inhibitor 2-deoxy-D-glucose.**

**
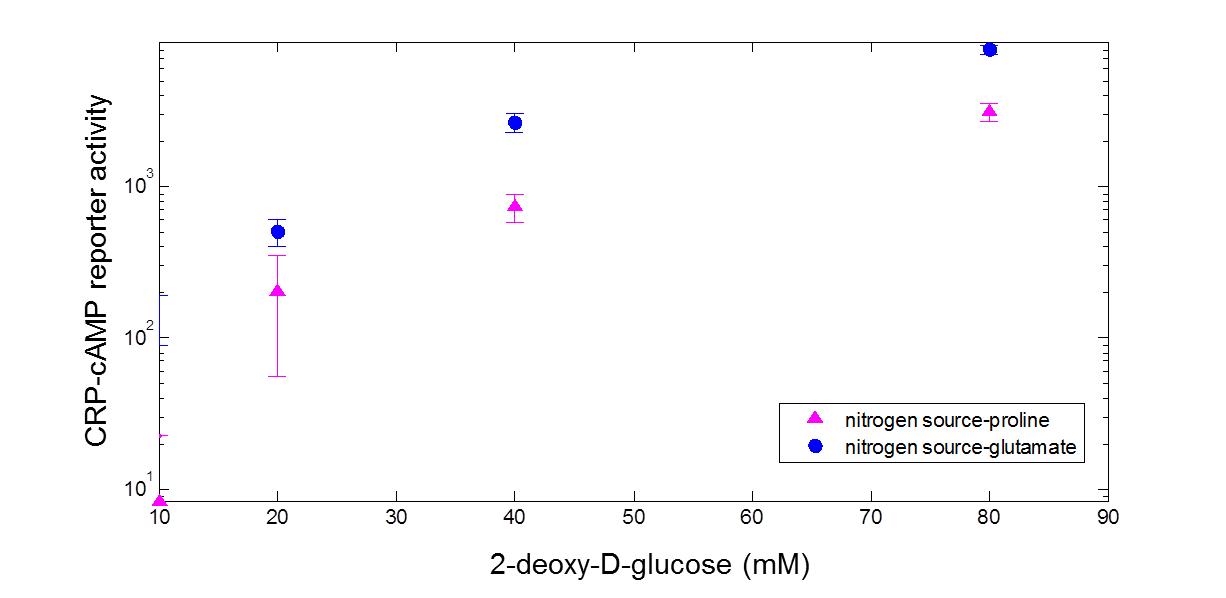
**

**Fig S10. CRR-cAMP levels increased in the presence of glucose uptake inhibitor.** Promoter activity of a synthetic CRP-cAMP reporter with different nitrogen sources and externally added 2-deoxy-D-glucose in the indicated levels. Each point is the average promoter activity and growth rate of 3 independent experiments with 6 experimental replicates in each experiment. Error bars represents the standard deviation of biological repeats.

***
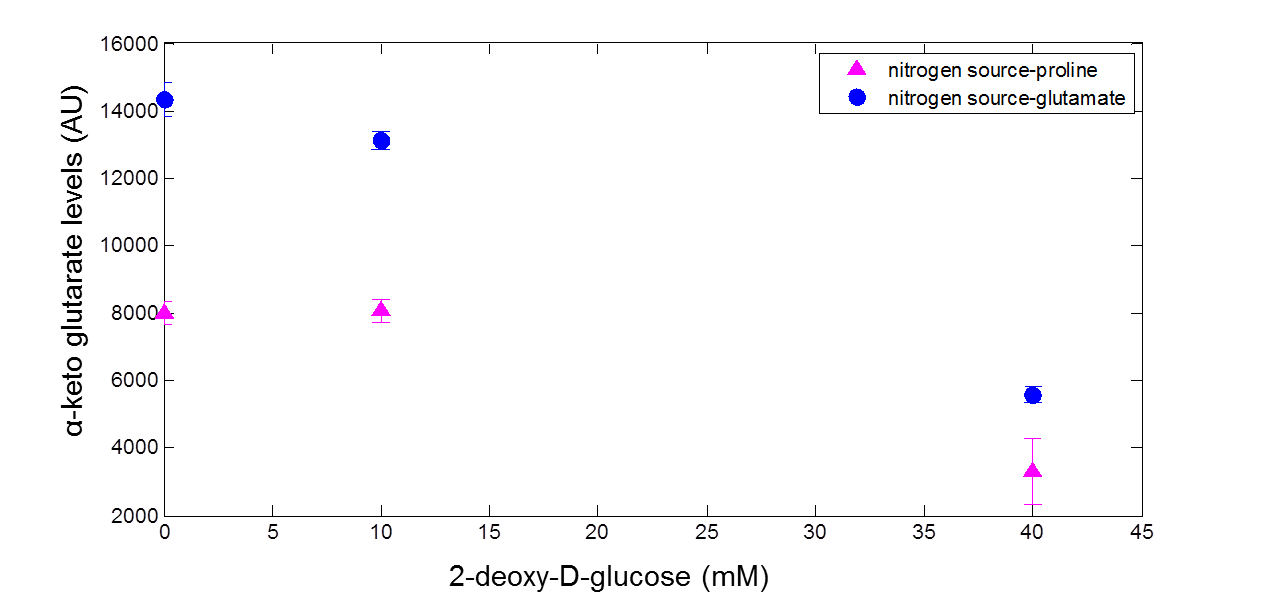
***

**Fig S11. αKG levels decreased with 2-deoxy-D-glucose.** Cells were grown in glucose (0.05%) + Proline or glutamate (10mM) and the indicated levels of 2-deoxy-D-glucose. αKG levels are ion counts normalized by OD and extracted culture volume. Metabolite extraction and analysis were performed as described in Methods.

1. **Growth on glucose and poor nitrogen source is significantly improved in a Δ*ptsG* strain**

**
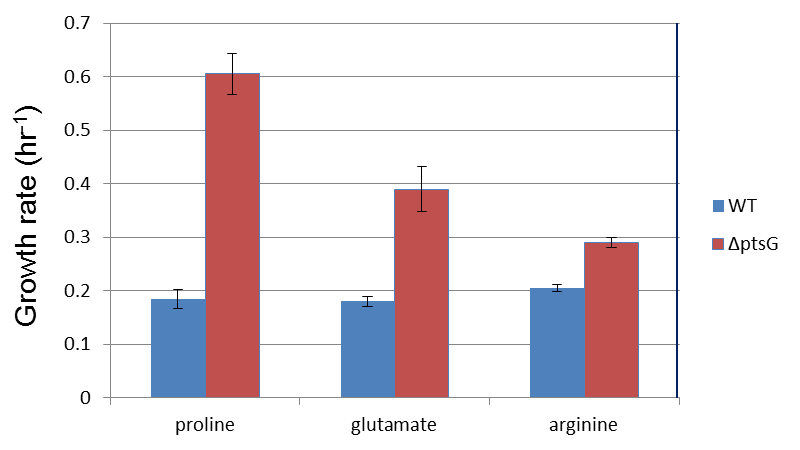
**

**Fig. S12. Growth on glucose and poor nitrogen sources is improve in a Δ*ptsG* strain.**

Exponential growth rate of NCM3722 WT or Δ*ptsG* strains in glucose (0.2%), with different nitrogen sources (10mM proline, glutamate or arginine).

1. **CRP-cAMP reporter shows a Michaelis-Menten- like response to external cAMP in a Δ*cyaA* strain.** As a control experiment, we tested the response of the CRP reporter used in this study to externally supplied cAMP in a strain that cannot synthesize cAMP (Δ*cyaA*). We found a Michaeli-Menten like response with a halfway point at around 2.5mM external cAMP.
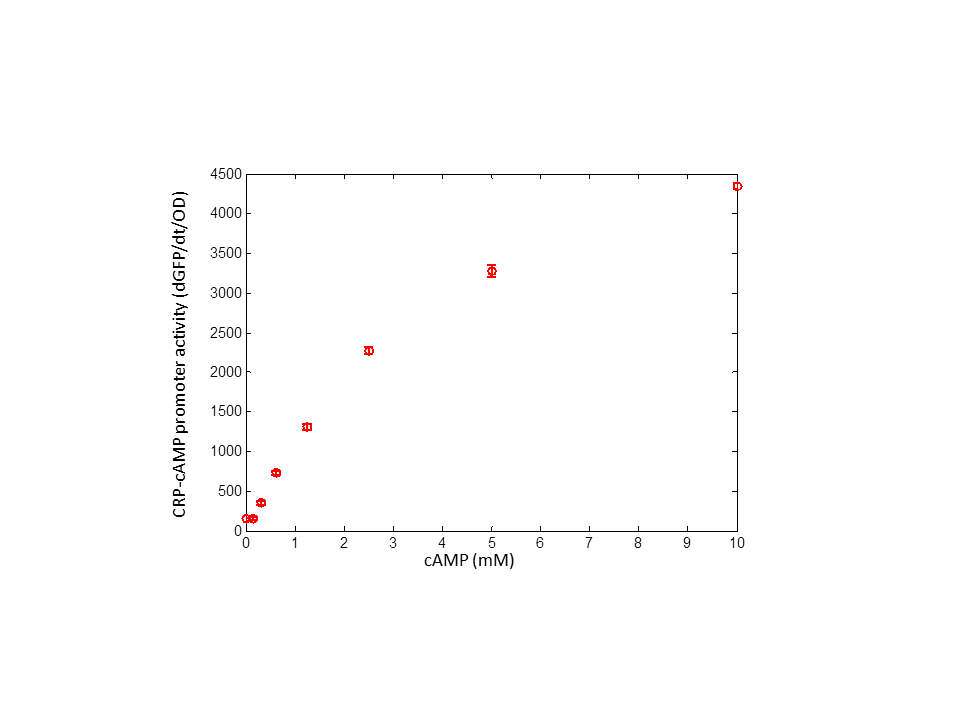
**Fig S13**. **Promoter activity of a CRP-cAMP synthetic reporter in a Δ*cyaA* strain grown in M9 minimal media with glucose as a carbon source, in the presence indicated levels of external cAMP**. OD and GDP measurments were obtained at a time resolutions of 8 min, averaged over 6 experimental replicas.

**References**

1. Souza, V., Rocha, M., Valera, A. & Eguiarte, L. E. Genetic Structure of Natural Populations of Escherichia coli in Wild Hosts on Different Continents. *Appl. Environ. Microbiol.* **65,** 3373–3385 (1999).

2. Shoval, O. *et al.* Evolutionary trade-offs, Pareto optimality, and the geometry of phenotype space. *Science* **336,** 1157–1160 (2012).

3. Bennett, B. D. *et al.* Absolute metabolite concentrations and implied enzyme active site occupancy in Escherichia coli. *Nat. Chem. Biol.* **5,** 593–599 (2009).
